# Reducing Sedation for Pediatric Thoracic CT Imaging Using Volumetric Target-mode EKG Gating

**DOI:** 10.1097/pq9.0000000000000779

**Published:** 2024-12-24

**Authors:** Summit H. Shah, Cody M. Young, Jessica Morrison, Margarita Chmil, Lynne Ruess, Rajesh Krishnamurthy

**Affiliations:** From the * Department of Radiology, Center for Clinical Excellence, Nationwide Children’s Hospital, Columbus, Ohio; † Center for Clinical Excellence, Nationwide Children’s Hospital, Columbus Ohio; ‡ Department of Radiology, The Ohio State University College of Medicine, Columbus, Ohio.

## Abstract

**Introduction:**

Many children require sedation for imaging. We aimed to reduce sedation for thoracic (chest and cardiac) computed tomography (CT) scans in children 0–4 years old from 65% to 20% by December 2018 and to sustain.

**Methods:**

We counted baseline, intervention, and a follow-up period thoracic CT scans performed with sedation in children 0–4 years old. We developed a new volumetric target-mode electrocardiogram-gated CT imaging protocol to reduce scan time and control for factors that decrease image quality. Additional interventions included technologist training, communication to radiologists and clinicians, and eliminating a default request for sedation accompanying the electronic order for most thoracic CT scans. A statistical process control chart tracked data to study process changes over time.

**Results:**

During the baseline and intervention periods, 232 of 357 and 217 of 794 scans required sedation. Interventions created 2 centerline shifts. Overall, thoracic CT scans in children 0–4 years old requiring sedation decreased from 65% to 24% and was sustained 5 years later. No patients during the baseline period, one (1 of 794, 0.1%) during the intervention period and 2 (2 of 480, 0.4%) during the audit period 5 years later, initially had nondiagnostic nonsedated scans that required an additional scan with sedation.

**Conclusions:**

We developed a volumetric target-mode electrocardiogram-gated CT protocol, eliminated default sedation ordering, and trained and educated staff to reduce sedation in young children undergoing thoracic CT scans. The frequency of sedation for thoracic CT in children 0–4 years old decreased from 65% to 24% and was sustained after revising imaging parameters and eliminating a default sedation order.

## INTRODUCTION

As pediatric imaging utilization has increased, so has the total number of sedations for pediatric imaging.^1^ In our practice, 65% of all thoracic computed tomographies (CTs) for cardiac, pulmonary and mediastinal indications in children less than 5 years required sedation. Advantages of sedation for imaging include a lower likelihood of diagnostic errors, fewer imaging quality concerns, and fewer incomplete reports.^2^

However, sedation use also carries higher cost, potential immediate complications, and suspected long-term effects.^3^ The availability of anesthesia resources is another limitation. Although serious events rarely occur, complications during or immediately after sedation may include respiratory, cardiovascular, or neurologic events.^3–7^ The US Food and Drug Administration reports that exposure to general anesthetic and sedation drugs for lengthy periods of time or over multiple procedures may negatively affect brain development in children younger than 3 years.^8,9^ Additional concerns related to sedation include hypoxia, delayed gastrointestinal complaints, restlessness, agitation, and motor imbalance.^7^ Furthermore, upper respiratory tract infection, history of obstructive sleep apnea or snoring, and obesity are all associated with failed sedation.^6^ Insufficient levels of sedation may pose risks including inadequate image quality and potential need for repeat examination and increased overall radiation doses.^10^

Reducing sedation through innovation is an important imaging goal. Efforts to achieve this goal must be balanced against the risk of diagnostic errors, poor image quality related to motion artifact, and additional radiation exposure related to repeat studies.^11,12^ Previous successful efforts to reduce sedation for pediatric imaging have included new imaging protocols and techniques, use of multidisciplinary teams including child-life specialists, judicious patient selection, and simulator training programs.^12–14^ Most available technology innovation literature focuses on reducing the higher sedation rates for magnetic resonance imaging (MRI), whereas few projects have focused on innovations to reduce anesthesia use for CT scans.^15^ We noted that the sedation rate for many types of CT examination had been decreasing in our practice, yet the sedation rate for thoracic CT scans remained high. Imaging the lung parenchyma with free-breathing is particularly challenging due to any motion compromising the fine detail needed to assess the small airways and pulmonary interstitium. Over the last few years, new generation CT using dual-source or volumetric technology have recently enabled motionless imaging of the chest in unsedated children.^16–19^ This article describes our experience with implementation of a program for reducing sedation for thoracic CT in children using volumetric CT technology.

### SMART Aim

Our quality improvement project aimed to reduce sedation for thoracic (chest and cardiac) CT scans in children 0–4 years old from 65% to 20% by December 2018 and to sustain the improvement overtime.

## METHODS

### Context

Our institution is a large urban pediatric quaternary care hospital. At the time of this project, we performed approximately 9000 CT scans each year with approximately 1800 thoracic CT scans. We formed a team of radiologists, CT technologists, and quality improvement specialists to work on this quality improvement project. Article preparation followed the Standards for Quality Improvement Reporting Excellence 2.0 guidelines.^20^

At baseline, we used a standard helical CT scan technique requiring 2–3 seconds of scan time or an ungated volumetric scan lasting 0.35 seconds for most scans of the chest in infants and young children. Although this is a short time, image quality will still be suboptimal if any breathing or gross patient motion occurs during image acquisition. For this reason, our routine was to sedate most children 0–4 years old for thoracic CT scans using general endotracheal anesthesia and breath-holding. When a clinician placed an order in the electronic medical record (EMR, Epic, Verona, Wis.), an autopopulated statement that sedation was required for the study based on the patient’s age would display. At our institution, sedation and monitoring for imaging are provided by the anesthesia service, except for intensive care unit patients whose care is provided by critical care providers. Nonsedated examinations are performed with the child free-breathing and wrapped in an immobilization blanket and without any coaching or breathing assistance.

### Interventions

**Fig. 1.**
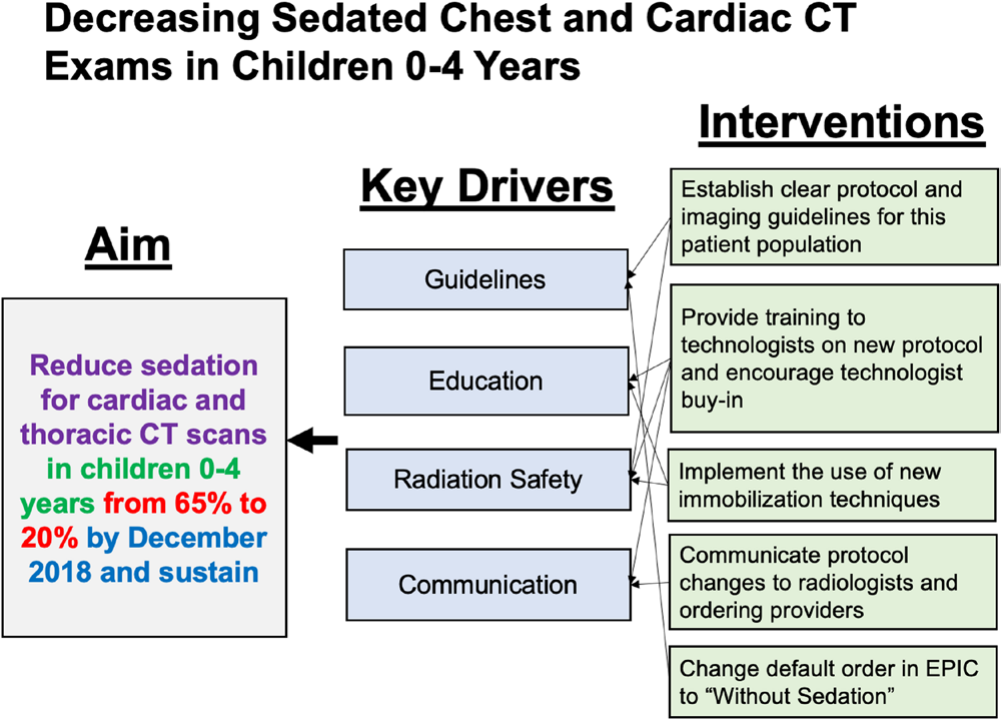
Key driver diagram outlines the aim, key drivers, and interventions for decreasing sedation rate for thoracic CT in children 0–4 years old.

The interventions are outlined in the key driver diagram (Fig. 1). For our first intervention, we developed a new CT imaging protocol aimed to reduce scan time and control for factors that decrease image quality related to respiratory, cardiac, and gross motion artifact. We used a 320-detector volumetric CT scanner (Aquilion One, Canon Medical Systems, Tustin, Calif.) and created a new target-mode electrocardiogram (EKG)-gated protocol.^19^ The volumetric acquisition is a single-revolution CT technique covering a maximum of 16 cm (whole chest coverage in children) with a scan time of 0.35 seconds. The protocol utilizes half-scan reconstruction to reduce the overall acquisition time to a fraction of the cardiorespiratory cycle, and target- mode prospectively EKG gating, which allows the use of a vendor product “Image Exact” application (Aquilion One, Canon Medical Systems) to reconstruct images that are free of cardiac and respiratory motion. The reconstruction is real time, and all images are immediately available for review after the scan. One caveat to note regarding the use of this technique for lung imaging is that the machine-picked motionless phase would be specific for cardiac motion. Therefore, specific attention to the lungs is needed to find an optimal reconstruction phase that specifically freezes lung motion, which might be different from the phase selected for cardiac motion.

We implemented routine use of the protocol for all thoracic CT scans in November 2016. Our second intervention was to train technologists to use the new protocol and make adjustments for respiration and choose a motionless phase of the cardiorespiratory cycle for the structure(s) of interest. Technologists used various immobilization techniques depending on patient age and the level of cooperation. Older and cooperative patients were simply swaddled in a sheet. Infants were swaddled in a sheet and used an additional device (9013 Toshiba CT Child Hugger with Table Adapter, Domico Med-Device, LLC, Fenton, MI). Children larger than the average 1 year old were swaddled in a sheet along with another device (Child Med-Vac, Domico Med-Device, LLC).

In July 2017, for our third intervention, we changed the default EMR order for thoracic CT scans to be a “without sedation” order. Between April and July 2018, for our fourth intervention, providers throughout our institution and radiologists were notified of this change via lectures and direct email communications.

### Study of Interventions

We retrospectively identified all thoracic CT (either chest CT or cardiac CT) examination in children 0–4 years old via EMRs (Epic Systems, Corp., Verona, Wis.) search. Image and medical record review identified sedated exams by the presence of endotracheal tube and/or anesthesia documentation. All patients with a tracheostomy tube were also considered as sedated.

The frequency of thoracic CT examination completed with sedation, or sedation rate, in children 0–4 years old was determined by dividing the number of examination completed with sedation (numerator) by the number of thoracic CT examination performed (denominator). We reviewed data monthly from the baseline period (January 2016–November 2016) through the intervention period (December 2016–December 2018). We also performed an ad hoc data audit during a 9-month follow-up period 5 years later (January 2023–September 2023).

A radiology quality coordinator loaded data into a proprietary statistical process control chart template, allowing the team to study process changes over time.^21^ We utilized the American Society for Quality (ASQ) criteria for adjusting the centerline and control limits for the statistical process control chart. In addition, we reviewed the process stages for variations. Annotated charts indicate the timing of interventions.

Because the tolerance for image quality issues varies based on indication, we used the radiologists’ assessment of diagnostic adequacy for a given indication to guide assessment of image quality, which was a separate field in the radiology report. For a balancing measure, we also tracked the number of repeat scans or appointments required for reimaging due to incomplete or nondiagnostic nonsedated CT.

### Ethical Considerations

This QI project involved implementing evidence-based interventions or best practices designed to reduce the use of sedation for chest CTs in children. Interventions did not involve multiple device comparisons or therapies, and patients were not subjected to randomization. This quality improvement work was exempt from institutional review board review per institutional policy.

## RESULTS

### Study Population

During the baseline period (January 2016–November 2016), 292 children underwent 357 thoracic CT scans (mean age at time of scan 1.4 years old, range 0–4 years old) (Table 1). During the intervention period (December 2016–December 2018), 626 children underwent 794 thoracic CT scans (mean age at time of scan 1.6 years old, range 0–4 years old). During the ad hoc follow-up period, 385 children underwent 480 thoracic CT scans (mean age at time of scan 1.3 years old, range 0–4 years old). The indications spanned the entire spectrum of thoracic and cardiac CT in children. Because consecutive studies were included, no indications were excluded. The commonest pulmonary indication groups for the studies were complicated pneumonia, oncological workup, HRCT, cystic fibrosis, and proximal airway assessment. Commonest cardiac indication groups included neonatal CHD, single ventricle pathway, complex 2 ventricle repair, aortopathy, and systemic and pulmonary venous pathology.

**Table 1.** Patient Age and Thoracic CT Examination Types in Children 0–4 Years during 3 Time Periods

Time period	All Thoracic CT, n, Mean Age, y	Sedated Thoracic CT, n (%), Mean Age, y	Sedated Thoracic CT Examination Type
Baseline (11 mo) January 2016–November 2016	357, 1.36	232 (65), 1.31	Chest 225 Cardiac 7
Intervention (2 y) December 2016–December 2018	794, 1.62	217 (27), 1.47	Chest 200 Cardiac 17
Follow-up (9 mo) January 2023–September 2023	480, 1.27	118 (25), 1.01	Chest 94 Cardiac 24

### Sedation Rates

**Fig. 2.**
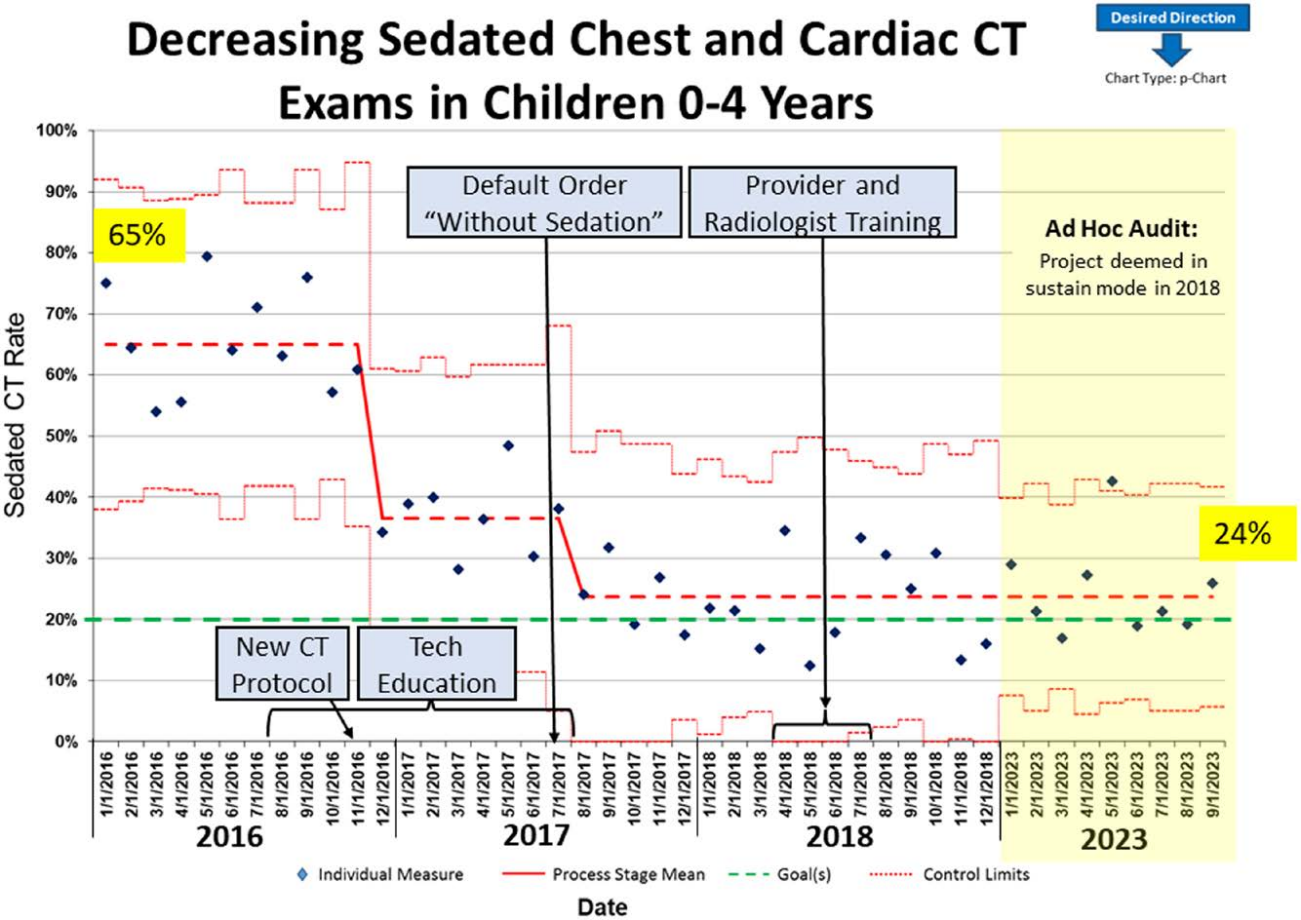
P-chart shows the sedation rate change for thoracic CT in Children 0–4 years old over time. Interventions are indicated in blue text boxes.

During the baseline period (January 2016–November 2016), thoracic CT sedation rate for children 0–4 years old was 65% (Fig. 2). After implementing interventions, significant improvement based on American Society For Quality criteria was evident.^22^ First, after implementing the volumetric imaging protocol, the centerline with associated control limits shifted to 37%. After establishing the “without sedation” clinician order default and educating providers and radiologists regarding nonsedated image acquisition options and techniques, the centerline shifted again to 24% and was maintained through December 2018 at which time the project entered sustain mode. Ad hoc data (January 1, 2023–September 30, 2023) audit 5 years later revealed unchanged sedation rates for thoracic CT in young children maintained at 24%.

### Balance Measure

All of the nonsedated scans during the baseline period were diagnostic for clinical indication. Five patients (5 of 626, 0.7%), during the intervention period had motion-degraded images and required more than one attempt to obtain diagnostic images during the initial nonsedate appointment. One (1 of 794, 0.1%) chest CT scan patient during the intervention period and 2 (2 of 480, 0.4%) cardiac CT scan patients during the audit period 5 years later, initially had nondiagnostic nonsedated scans which required an additional scan with sedation. During the intervention period, a 2-year old returned for a second appointment completed with sedation. During the follow-up period, a 4-month old infant had an appointment for thoracic CT with sedation for interstitial lung disease, but the examination was attempted without sedation first. Motion degraded the images and the infant was sedated and a diagnostic scan was completed at the same appointment. Also during the follow-up period, a 4-year old was inconsolable at the time of attempted nonsedate scan and was rescheduled with sedation.

## DISCUSSION

We significantly decreased pediatric thoracic CT sedation rates in our practice using a volumetric, target-mode EKG-gated protocol, eliminating a default EMR sedation order for children 0–4 years old, and by educating and training staff. We achieved and sustained a reduction in our sedation rate from 65% to 24% while maintaining diagnostic quality examinations. During the 2-year intervention period, we eliminated sedation for 557 examinations in children (0–4 years old), and on follow-up 5 years later, most of the thoracic CT exams in young children continue to be successfully performed without sedation. We believe these results can be replicated and implemented by other pediatric facilities utilizing the volumetric target-mode imaging protocol and careful attention to clinician exam ordering options.

Regarding the technical innovation on this study, the “target-mode” prospectively EKG-gated technique and the “ImageExact” techniques are approved products offered by the vendor, with similar offerings from other makers of the volumetric technique of CT. Our group has previously published on its use for motionless imaging of the heart for congenital heart disease^19^ An innovation of this study was in its use to reduce respiratory motion artifact, not only for cardiac imaging, but also for lung and airway imaging, which has not been reported previously. The only additional customization needed is adding EKG leads and turning on target-mode EKG gating during lung CT acquisition, similar to the process used for cardiac CT. Once the acquisition is complete, the technologist would use the “ImageExact” technique to perform half-scan reconstruction of a motionless phase of the cardiorespiratory cycle and submit it for interpretation by the radiologist.

Our goal was to decrease sedation rate to 20%, recognizing that there are certain indications for which young children might still require sedation for thoracic CT imaging. We expect to continue to use sedation for intensive care patients who are already sedated, and for indications that require controlled inspiration and expiration to screen for air-trapping for instance because precise respiratory gating is not available with the target-mode technique. Also, some children may be scheduled for sedation to facilitate multiple exams or procedures in one day. We are exploring the use of respiratory gating techniques that may be combined with the target-mode EKG gating to further reduce the current sedation rate of 24% in this population.

We also identified some system barriers to reducing sedation rates. Some young children eligible for nonsedated imaging continued to undergo thoracic CT with sedation during the intervention period. There were several reasons for this. First, some of the radiologists and referring clinicians preferred or were accustomed to the default sedation pathway and older CT protocols which provided excellent imaging under general endotracheal anesthesia, and continued to order the use of sedation for chest CT even after several educational sessions presenting data on the efficacy of the new volumetric protocol. Once scheduled with sedation, the CT workflow proceeded efficiently before an unsedated alternative could be suggested by the technologist or radiologist on duty. Anecdotal evidence suggested that these barriers decreased with education and with increased comfort level with the comparable quality of the new CT protocol. With time, technologists recognized which children might be eligible and intervened to change the protocol before the patient’s arrival for the scan. In addition to education, changes to the sedation order entry system in the EMR were necessary to enact a further meaningful drop in sedation rates. Thus, this project demonstrated the importance of incorporating additional tools needed to successfully implement a technical innovation and effect long-term change. Although education and training was important, changing the structure with the new CT protocol and the process with the new default order for unsedated CT imaging were the likely key drivers of the center line shifts and for the sustainability of our results. Changing the default order guided and encouraged clinicians to select the appropriate study as clinical decision support tools tend to do. Previous studies have affirmed this by showing that changing the default orders in the EHR can change behaviors of ordering providers in the pediatric setting and guide medical decision making.^23,24^ These studies have further encouraged hospital systems to implement more expert-recommended defaults to EHR orders.^24^

A concern for performing a thoracic CT in a young child without sedation is that it will result in a clinically inadequate study and will require repeat imaging with or without sedation necessitating additional appointments, further cost, and potential for increased radiation exposure. A major driver for adoption of the unsedated image acquisition approach by referring clinicians and radiologists was the sharing of data on comparable image quality at several venues including educational lectures, clinical care meetings and regional and national scientific meetings. After implementing the target-mode EKG-gated protocol for nonsedated thoracic CT, image quality was deemed to be adequate by the interpreting body radiologists for more than 99% of thoracic CT exams. In addition, an added advantage of unsedated imaging was the lack of dependent atelectasis that was frequently encountered with general endotracheal anesthesia.^25^

Limitations of the study include the fact that this is a retrospective study spanning many years based on review of the patient’s electronic health record. Imaging evidence of airway manipulation was used to augment the EHR sedation records in the baseline and postintervention period. Although anesthesia records for imaging sedation were readily available, there was a small subset of patients who were typically intubated or ventilated via a tracheostomy for their underlying condition, and might have underwent breath-holding for the imaging study in the baseline period with supervision provided by the ICU team. In the postintervention period, these patients were likely performed without any additional sedation or breath-holding. Our recording system or the patient’s EHR did not track respiratory manipulation performed by the ICU team in the radiology suite. We made the decision to include every patient with an indwelling airway tube in the sedation group to maintain consistency between the baseline and postintervention period. This likely resulted in a small artifactual increase in the sedation rate in the postintervention period.

From a scaling perspective, a robust QI infrastructure may not be available to support similar projects at all institutions. Some practices may not have the necessary equipment and technology to implement the target-mode EKG-gated volumetric CT image acquisition protocol. However, other technologies such as dual-detector image acquisition may offer similar scan speeds to achieve similar results.^17,18^ Other studies have shown similar results reducing sedation and reducing scan times using volume CT protocols.^26,27^ Additionally, savings on institutional resources such as anesthesia and greater throughput may allow for investments into new CT technology. We did not control for any patient factors or examine the impact of different disease states on the rate of sedation or image quality. We also did not study the positive impact of reducing the need for sedation on the use of highly skilled resources like anesthesia, the degree of improvement in system inefficiencies like scheduling lag or exam turnaround time, or potential cost-savings to the system.

Decreasing the need for sedation for diagnostic purposes in the pediatric population is an important priority. Not only does this goal decrease the probability of immediate and potential long-term complications of sedation use but also has the potential to reduce system inefficiencies, costs, and utilization of highly skilled resources.

## CONCLUSIONS

Leveraging advances in imaging technology and updating clinical practice patterns are complementary means to improve pediatric healthcare. We developed a volumetric target-mode EKG-gated CT protocol, eliminated default sedation ordering, and educated and trained staff to significantly reduce sedation in young children undergoing thoracic CT scans.

## References

[1] Wachtel RE Dexter F Dow AJ. Growth rates in pediatric diagnostic imaging and sedation. Anesth Analg. 2009;108:1616–1621. 10.1213/ane.0b013e3181981f96 19372345

[2] Stern KW Gauvreau K Geva T. The impact of procedural sedation on diagnostic errors in pediatric echocardiography. J Am Soc Echocardiogr. 2014;27:949–955. 24930122 10.1016/j.echo.2014.04.024 PMC4149941

[3] Cote CJ Karl HW Notterman DA. Adverse sedation events in pediatrics: analysis of medications used for sedation. Pediatrics. 2000;106:633–644. 11015502 10.1542/peds.106.4.633

[4] Malviya S Voepel-Lewis T Prochaska G. Prolonged recovery and delayed side effects of sedation for diagnostic imaging studies in children. Pediatrics. 2000;105:E42. 10699144 10.1542/peds.105.3.e42

[5] Kiringoda R Thurm AE Hirschtritt ME. Risks of propofol sedation/anesthesia for imaging studies in pediatric research: eight years of experience in a clinical research center. Arch Pediatr Adolesc Med. 2010;164:554–560. 20530306 10.1001/archpediatrics.2010.75 PMC3197223

[6] Grunwell JR McCracken C Fortenberry J. Risk factors leading to failed procedural sedation in children outside the operating room. Pediatr Emerg Care. 2014;30:381–387. 24849275 10.1097/PEC.0000000000000143

[7] Malviya S Voepel-Lewis T Tait AR. Pentobarbital vs chloral hydrate for sedation of children undergoing MRI: efficacy and recovery characteristics. Paediatr Anaesth. 2004;14:589–595. 15200658 10.1111/j.1460-9592.2004.01243.x

[8] Administration USFaD. FDA Drug Safety Communication: FDA approves label changes for use of general anesthetic and sedation drugs in young children. Available at https://www.fda.gov/drugs/drug-safety-and-availability/fda-drug-safety-communication-fda-approves-label-changes-use-general-anesthetic-and-sedation-drugs2017. Accessed January, 2024.

[9] McCann ME de Graaff JC Dorris L; GAS Consortium. Neurodevelopmental outcome at 5 years of age after general anaesthesia or awake-regional anaesthesia in infancy (GAS): an international, multicentre, randomised, controlled equivalence trial. Lancet. 2019;393:664–677. 30782342 10.1016/S0140-6736(18)32485-1 PMC6500739

[10] Arlachov Y Ganatra RH. Sedation/anaesthesia in paediatric radiology. Br J Radiol. 2012;85:e1018–e1031. 22898157 10.1259/bjr/28871143 PMC3500799

[11] Daldrup-Link HE Sammet C Hernanz-Schulman M. White paper on P4 concepts for pediatric imaging. J Am Coll Radiol. 2016;13:590–597.e2. 26850380 10.1016/j.jacr.2015.10.028 PMC4860067

[12] Ahmad R Hu HH Krishnamurthy R. Reducing sedation for pediatric body MRI using accelerated and abbreviated imaging protocols. Pediatr Radiol. 2018;48:37–49. 29292482 10.1007/s00247-017-3987-6

[13] Runge SB Christensen NL Jensen K. Children centered care: Minimizing the need for anesthesia with a multi-faceted concept for MRI in children aged 4-6. Eur J Radiol. 2018;107:183–187. 30292264 10.1016/j.ejrad.2018.08.026

[14] Carter AJ Greer ML Gray SE. Mock MRI: reducing the need for anaesthesia in children. Pediatr Radiol. 2010;40:1368–1374. 20186541 10.1007/s00247-010-1554-5

[15] Kaste SC Young CW Holmes TP. Effect of helical CT on the frequency of sedation in pediatric patients. AJR Am J Roentgenol. 1997;168:1001–1003. 9124104 10.2214/ajr.168.4.9124104

[16] Kino A Zucker EJ Honkanen A. Ultrafast pediatric chest computed tomography: comparison of free-breathing vs. breath-hold imaging with and without anesthesia in young children. Pediatr Radiol. 2019;49:301–307. 30413857 10.1007/s00247-018-4295-5

[17] Rapp JB Ho-Fung VM Ramirez KI. Dual-source computed tomography protocols for the pediatric chest—scan optimization techniques. Pediatr Radiol. 2023;53:1248–1259. 35948645 10.1007/s00247-022-05468-7 PMC9365683

[18] Tivnan P Winant AJ Johnston PR. Thoracic CTA in infants and young children: image quality of dual-source CT (DSCT) with high-pitch spiral scan mode (turbo flash spiral mode) with or without general anesthesia with free-breathing technique. Pediatr Pulmonol. 2021;56:2660–2667. 33914408 10.1002/ppul.25446

[19] Jadhav SP Golriz F Atweh LA. CT angiography of neonates and infants: comparison of radiation dose and image quality of target mode prospectively ECG-gated 320-MDCT and ungated helical 64-MDCT. AJR Am J Roentgenol. 2015;204:W184–W191. 25615779 10.2214/AJR.14.12846

[20] Goodman D Ogrinc G Davies L. Explanation and elaboration of the SQUIRE (Standards for Quality Improvement Reporting Excellence) Guidelines, V.2.0: examples of SQUIRE elements in the healthcare improvement literature. BMJ Qual Saf. 2016;25:e7. 10.1136/bmjqs-2015-004480 PMC5256235 27076505

[21] Benneyan JC Lloyd RC Plsek PE. Statistical process control as a tool for research and healthcare improvement. Qual Saf Health Care. 2003;12:458–464. 14645763 10.1136/qhc.12.6.458 PMC1758030

[22] American Society for Quality. Quality resources—control chart. 2021. Available at https://asq.org/quality-resources/control-chart. Accessed April 5, 2021.

[23] Barry C Kaufman S Feinstein D. Optimization of the order menu in the electronic health record facilitates test patterns consistent with recommendations in the choosing wisely initiative. Am J Clin Pathol. 2020;153:94–98. 31433839 10.1093/ajcp/aqz134

[24] Probst CA Shaffer VA Chan YR. The effect of defaults in an electronic health record on laboratory test ordering practices for pediatric patients. Health Psychol. 2013;32:995–1002. 24001250 10.1037/a0032925

[25] Sargent MA McEachern AM Jamieson DH. Atelectasis on pediatric chest CT: comparison of sedation techniques. Pediatr Radiol. 1999;29:509–513. 10398785 10.1007/s002470050632

[26] Zhu Y Li Z Ma J. Imaging the infant chest without sedation: feasibility of using single axial rotation with 16-cm wide-detector CT. Radiology. 2017;286:279–285. 28956735 10.1148/radiol.2017170019

[27] Kroft LJ Roelofs JH Geleijns J. Scan time and patient dose for thoracic imaging in neonates and small children using axial volumetric 320-detector row CT compared to helical 64-, 32-, and 16-detector row CT acquisitions. Pediatr Radiol. 2010;40:294–300. 19997730 10.1007/s00247-009-1436-x PMC2817802

